# Heroin Inhalation Leukoencephalopathy: An Overlooked Entity in the Opioid Epidemic

**DOI:** 10.7759/cureus.40535

**Published:** 2023-06-16

**Authors:** Aidan Farrell, Benjamin O'Brien, Patrick Janeczko, Nicholas Cassimatis, David Monoky

**Affiliations:** 1 Radiology, Hackensack Meridian School of Medicine, Nutley, USA; 2 Neurological Surgery, Hackensack University Medical Center, Hackensack, USA; 3 Radiology, Hackensack University Medical Center, Hackensack, USA

**Keywords:** altered mental state, neuroradiology, opioid epidemic, toxic encephalopathy, heroin inhalation

## Abstract

Heroin inhalation leukoencephalopathy (HIL) is a rare complication of vaporized heroin inhalation leading to white matter degeneration resulting in a range of neurologic disturbances including softened speech, cerebellar ataxia, behavioral changes, cerebellar gait abnormalities, and even respiratory failure resulting in death in the most severe cases. Magnetic resonance imaging (MRI) most commonly demonstrates bilateral hyperintensities affecting the basal ganglia, periventricular white matter, and cerebellum. In this case report, we present a relatively mild case of HIL in a young female patient to describe the characteristic challenges associated with the condition’s presentation, diagnosis, and treatment. While healthcare workers everywhere are addressing a complex and ever-changing opioid epidemic, we strive to raise awareness about HIL as only one of a variety of complications resulting from opioid use disorder.

## Introduction

Over half a million Americans died of opioid-related drug overdose from 1999 to 2020 [1]. The appearance of illicit black-market fentanyl in the late 2010s has only accelerated the increases in the rates of morbidity and mortality [2]. In March of 2023, the Food and Drug Administration, in response to staggering numbers of deaths, approved the opioid antagonist naloxone for over-the-counter purchase in order to equip more Americans with the tools to save the lives of those undergoing a drug overdose [3]. Furthermore, a plethora of different government-funded campaigns, initiatives, and research seeks to glean new insights and educate healthcare providers, drug users, the broader public, and other stakeholders about what has become the greatest threat to public health of this century. Despite this, a comprehensive understanding of opioid use disorder and associated pathologies remains poor.

An example of this is heroin inhalation leukoencephalopathy (HIL), a rare disorder that causes degeneration of the white matter of the brain and spinal cord that is caused by the inhalation of vaporized heroin fumes. This condition is of particular concern because many people, drug users and the general public alike, believe that smoking is a safer alternative, both in terms of risk of drug overdose and the contraction of bloodborne diseases, to intravenous injection [4]. The exact pathogenesis of HIL is unknown but is thought to be caused by the generation of toxic lipophilic substances during the heating process that deposits in white matter [5]. In the initial stages, HIL presents with non-focal signs such as restlessness, agitation, and changes in behavior. Progression of HIL is characterized by hypotonic paresis and spasms, likely due to bilateral posterior limb of the internal capsule and corticospinal tract involvement, as well as slowed processing. Additionally, akinetic mutism related to bilateral hemispheric involvement may be present. The progression of these symptoms may result in severe disability and death. Diagnosis involves conscientious history taking to confirm the use of heroin by inhalation, a careful inventory of patient symptoms, and the recognition of characteristic imaging findings. These imaging findings include bilateral hyperintensities in the posterior limbs of the internal capsules, periventricular white matter, and the cerebellum on T2-weighted and fluid-attenuated inversion recovery (FLAIR) magnetic resonance imaging (MRI) [6,7].

## Case presentation

A 25-year-old woman with no past medical history presented to the emergency department with a chief complaint of slurred speech for four days. The patient also related difficulties with handwriting. The patient stated that symptom onset occurred suddenly while she was at work four days ago. Symptoms were described as episodic, lasting between 15 minutes and four hours, before remitting during which the patient had trouble “getting words out” or writing on a piece of paper with a pen. The patient was experiencing several of these kinds of episodes a day. Symptom quality and severity stayed constant since symptom onset and the patient could not identify any worsening or alleviating factors. The patient denied all other symptoms including fever, chills, focal weakness, numbness, tingling, headaches, difficulty ambulating, nausea, emesis, chest pain, dyspnea, and any other complaints. Her physical examination, including neurological examination, was non-focal and within normal limits. She adamantly denied using any medications or illicit drugs. She endorsed smoking half a pack of cigarettes for the past 10 years.

Initial differential diagnosis was broad and included but was not limited to intracranial mass, multiple sclerosis, toxic leukoencephalopathy, other demyelinating diseases of the central nervous system, electrolyte imbalance, anxiety, and functional disorder. The initial workup consisted of a comprehensive metabolic panel, a complete blood count, and a computed tomography (CT) scan of the head without intravenous contrast.

The comprehensive metabolic panel showed a mild elevation in liver transaminases which was thought to be unrelated to the patient’s presentation. The complete blood count showed no abnormalities.

**Figure 1:**
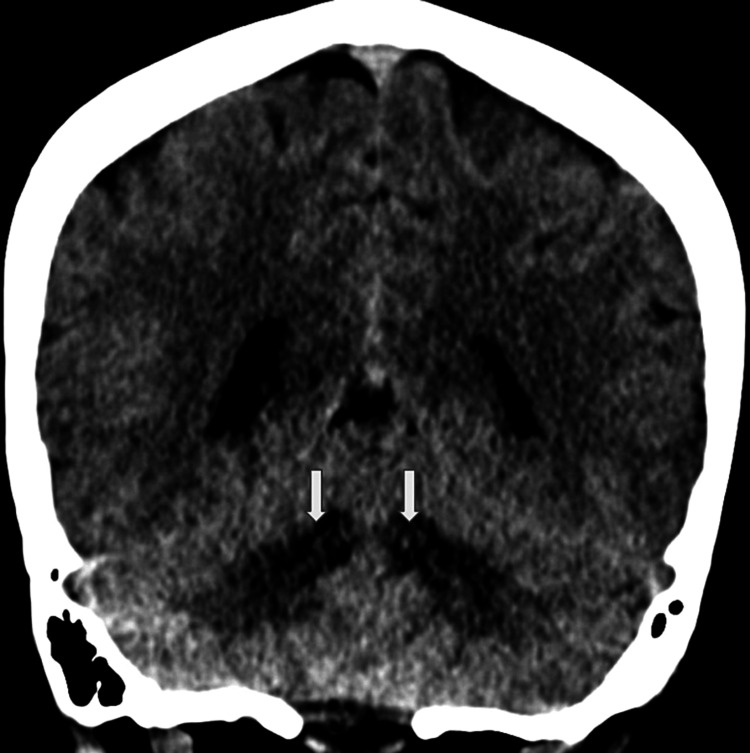
CT coronal reconstruction demonstrating abnormal symmetric cerebellar low densities (indicated by arrows).

**Figure 2:**
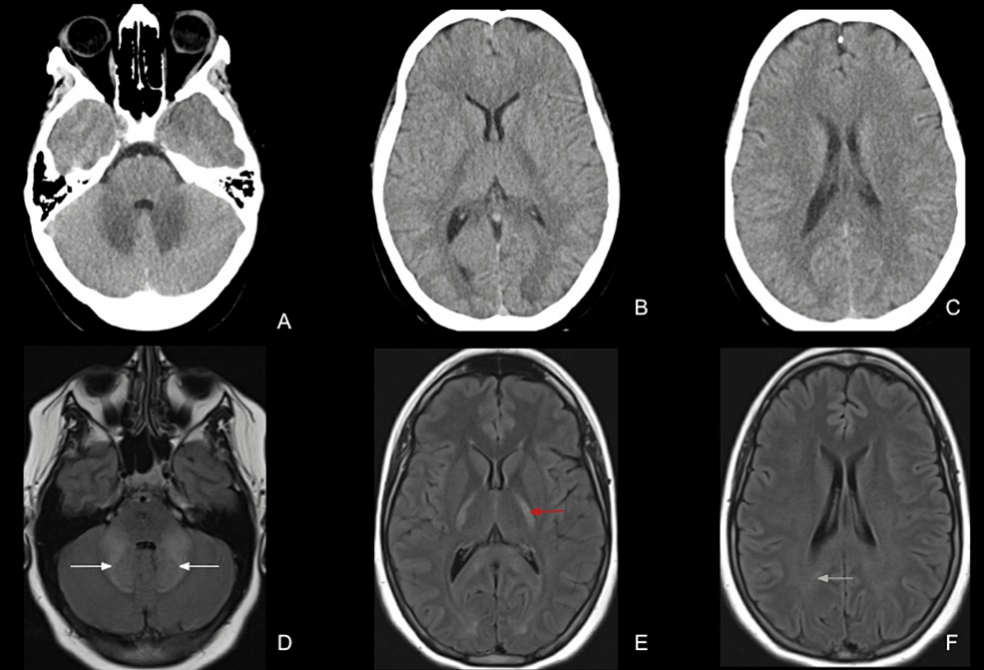
Top row: Axial CT scan of the head without contrast at the level of the cerebellum (A), internal capsule (B), and lateral ventricles (C), respectively. Bottom row: Accompanying axial FLAIR MRI at the level of the cerebellum (D), internal capsule (E), and lateral ventricles (F). Symmetric abnormal bright signals (indicated by arrows) are noted in the cerebellar peduncles (D) (white arrows), posterior limb of the internal capsule (E) (red arrow), and the occipital periventricular white matter (F) (yellow arrow).

CT imaging of the head revealed fairly symmetric abnormal low attenuation involving the brachium pontis and cerebellar white matter tracts, and more subtly, within the posterior limbs of the internal capsules bilaterally (Figures 1, 2A-2F). The specific etiology of these changes was uncertain, so a broad range of differential diagnoses was considered. Toxic and metabolic etiologies considered included heroin inhalation toxicity, solvent inhalation toxicity, and metronidazole toxicity. Inflammatory demyelinating processes such as neural myelitis optic (NMO) and acute demyelinating encephalomyelitis (ADEM) were also included in the differential.

Overnight admission to the hospital for a magnetic resonance scan of the brain was recommended. However, the patient insisted that she did not want to stay and would follow up at an outpatient neurology office for further evaluation. After being informed of the possible risks of this decision, the patient signed out against medical advice (AMA).

Three days later, the patient returned to the emergency department because of the persistence of symptoms and was admitted to the internal medicine service. The patient’s grandmother noted that she has seemed more withdrawn and less talkative since symptom onset. Neurology was consulted and agreed with the plan for imaging and further workup for demyelinating disease. MR imaging of the brain with and without contrast was then pursued.

MRI revealed abnormal bilateral T2/FLAIR hyperintensities involving the posterior internal capsules, brachium pontis, and cerebellar hemisphere white matter tracts, and along the occipital periventricular white matter (Figures 2A-2F). MRI of the spine showed no evidence of demyelinating disease. These findings were highly concerning and suspicious for an underlying toxic-metabolic leukoencephalopathy and prompted an extensive discussion regarding the use of illicit drugs, particularly industrial solvents and inhaled heroin, which the patient adamantly denied. Accordingly, it was decided to pursue a workup for demyelinating disease by obtaining an MRI of the cervical and thoracic spine, lumbar puncture, and additional blood work. A urine toxicology screen was obtained at that time as the neuroradiologist favored a diagnosis of toxic leukoencephalopathy over demyelinating disease. Additionally, a course of high-dose steroids was initiated at that time.

One day later, the patient underwent an MRI of the cervical and thoracic spine which were unremarkable. Shortly after, the patient’s mother approached the neurologist and told him she suspected the patient of using heroin. When asked why she held these suspicions, the patient’s mother stated that she found small plastic baggies containing a powder in the patient’s room. When the neurologist asked the patient about this, the patient apprehensively admitted that she was, in fact, using heroin. Furthermore, the patient stated that she had never injected heroin intravenously and instead inhaled vaporized heroin fumes, a process known colloquially as “chasing the dragon.”

The patient was diagnosed with HIL and was started on coenzyme Q 300 mg four times daily, vitamin E 2000 mg once daily, and vitamin C 2000 mg once daily. Addiction psychiatry, social work, child services (as the patient was the lone caretaker of an infant), physical therapy, and occupational therapy were consulted. The next morning the patient’s nurse noticed that the patient had hidden a nicotine vaporizer in her room. After being informed that nicotine vaporizers are not allowed in the patient rooms, the patient became agitated and began demanding to leave. Despite hospital staff trying to calm her down, the patient became more upset and was escorted out by hospital security and was lost to follow-up.

## Discussion

Heroin inhalation is an uncommon, but important cause of encephalopathy to consider in young patients regardless of documented history of drug use. HIL can be broken down into three unique stages. The initial stage, lasting for two to four weeks, is characterized by motor restlessness, soft (pseudobulbar) speech, cerebellar ataxia, and apathy [8]. Around half of HIL patients will then progress to the intermediate stage, where rapid progression of symptoms is seen, particularly cerebellar gait disturbances. Additional symptoms that begin during the intermediate stage include pyramidal tract signs such as hyperreflexia, and spastic paresis, in addition to tremors, and choreo-athetoid movements. Approximately 25% of patients will reach the terminal stage of HIL after an illness course of eight to 12 weeks, which usually results in death. Stretching spasms, hypotonic paresis, akinetic mutism, and central pyrexia are the symptoms associated with terminal-stage HIL [8]. Death in this stage is most commonly due to respiratory failure caused by hypotonic paresis. The patient in this report was most likely in the initial stage of HIL, which is supported by her dysarthria, other signs of motor dysfunction, and the onset of symptoms four days before her presentation to the hospital.

Patients with HIL may be obtunded or uncooperative making accurate history difficult, so MRI is important in diagnosis. Classically described MRI findings in the setting of HIL include symmetric and confluent T2/FLAIR hyperintensities within the cerebellar white matter, cerebellar peduncles, and posterior limbs of the internal capsules, all of which were present on the patient’s scan [9]. It is important to note that imaging findings may worsen, even when patients are showing signs of clinical improvement.

 HIL falls under the broader diagnosis of acute toxic leukoencephalopathy (ATL). The different causes of ATL generally share common imaging findings of bilateral symmetric confluent areas of diffusion restriction in the periventricular white matter, with cortical gray matter, deep gray nuclei, thalami, and the corpus callosum also being susceptible to these abnormalities [6]. ATL is related to excitotoxic brain injury, with areas of higher susceptibility being more prone to excitotoxic glutamate release [6]. More specific imaging findings and a comprehensive history and physical are crucial for differentiating HIL from other forms of ATL. The characteristic findings of HIL include a symmetric high signal in the posterior limb of the internal capsule and the cerebellum on T2 and FLAIR MR imaging [7]. Key imaging findings related to other conditions considered in our patient include involvement of the putamina and sparing of the globi pallidi seen in methanol poisoning, symmetric globi pallidi involvement in carbon monoxide poisoning, significant T2 hypointensity involving the thalami, basal ganglia, and substantiae nigrae in solvent inhalation and asymmetric white matter involvement suggestive of demyelinating disease [6].

There is no established treatment for HIL, though antioxidant therapy is typically employed [10]. Clinical improvement has been reported in previous cases where antioxidant therapy was used, which supports the hypothesis of mitochondrial dysfunction having a role in the development of HIL [10]. The patient in this report was treated with coenzyme Q, vitamin C, and vitamin E though her ultimate clinical outcome is unknown to us.

It is essential to recognize that the diagnosis and treatment of this patient were delayed because of psychosocial factors surrounding the use of recreational drugs, especially opioids. The patient provided inaccurate history to healthcare providers throughout the course of her presentation and repeatedly and adamantly denied any history of drug use. Furthermore, the patient’s mother did not verbalize her suspicion of drug use until four days after the initial presentation.

Illicit drug use disorders are some of the most stigmatized health conditions in America [11]. The reasons for this stigma are multifactorial. Patients suffering from these conditions are often seen as difficult patients that are weak, bad, dangerous, or unworthy of receiving help. Additionally, many people blame illicit drug use disorder on moral weakness rather than viewing it as a chronic illness. This is demonstrated by the slow adoption of opioid agonist therapy or safe consumption centers by American public health policy [11]. Additionally, healthcare providers, who often treat individuals with opioid use disorder and other similar conditions, are also not immune from thinking in this way or possessing biases that negatively impact care.

Patients who suffer from opioid use disorder have their own reasons for not disclosing this part of their life. They may feel intense shame or apprehension at the possibility of having to admit they suffer from a highly stigmatized condition that can have genuine social and legal repercussions. They may be in a state of denial. They may be so deep in the throes of addiction that they may perceive engaging with the healthcare system as an endeavor that limits their access to the illicit substances, they are dependent upon. All of these possibilities are highly distressing. The various difficulties that these individuals face must be recognized and addressed effectively to ensure proper diagnosis and treatment.

## Conclusions

The opioid epidemic is one of the most significant threats to public health since the turn of the century, especially among the younger population, leading to tragic rates of morbidity and mortality. Persistent stigma surrounding opioid use disorder including efficacious treatment with medication-assisted treatment increases the risk for long-term complications due to illicit opioid use. Though HIL is a relatively infrequent sequela of inhaled heroin, this case demonstrates the importance of having a low diagnostic threshold for further investigation into substance use when a patient presents with typical clinical features such as speech changes and cerebellar ataxia when they are paired with suggestive radiologic findings. Early recognition of complications such as HIL provides healthcare providers the opportunity to intervene at the individual level to provide appropriate medical therapy and access to harm reduction resources to those suffering from opioid addiction.
